# 

**Published:** 1874-11

**Authors:** 


### MERRIMACK VALLEY DENTAL ASSOCIATION.

The Twelfth Annual Meeting of the Association will be held in Plummer Institute Hall, Essex Street, Salem, Mass., Nov. 5th and 6th.

Free Return Tickets are expected from the Eastern R. R

ESSAYISTS:

Dr. G. F. Waters, Mass.; "The Replanting and Transplanting of Teeth."

Dr. C. G. Davis, New Bedford, Mass.; "Poison by Amalgam Fillings."

Dr. I. J. Wetherbee, Boston Mass.; "Dental Education."

Volunteer Essays may be expected.

CLINICAL OPERATORS:

Drs. I. A. Salmon, D. B. Ingalls, and A. M. Dudley.

SUBJECTS FOR DISCUSSION:

Etiology of Dental Caries. Ought we to use Red Rubber? Miscellaneous Subjects.

Hotel Head-quarters at Essex House.

A. M. Dudley. Secretary.

There is also an appendix, containing valuable information in the way of practical suggestions, formulas, etc. The work we regard as a very valuable one to the student, and indeed for the practitioner. It should be in every dentist's library. It is reprinted in this country by Lindsay & Blakiston, and may be obtained of any bookseller who keeps medical work, price 2.50

### OHIO STATE DENTAL SOCIETY.

The annual meeting of the Ohio State Dental Society will be held in Columbus, O., in the City Council Chamber, on Wednesday, Dec. 2d, at 10 o'clock a. m., as will be seen by the notice below. We hope and believe that this will be the largest and most interesting meeting of this Society ever held and it is very desirable, that every one should come prepared to add something of interest and profit. The programme presents quite a number of subjects of great interest and practical importance.

Let every one who expects to be present bestow some attention and study upon each of the subjects, and so come better prepared both to teach and be taught, to give and receive. For all who make sacrifice to attend these meetings there are manifold rewards in store.

A mistake was made in the circular sent out by the Secretary of the Society as to the time of meeting of the Board of Dental Examiners. That meeting will take place on Tuesday, Dec. 1st, (instead of 2d as upon the circular) at 12 o'clock m. Let all who are interested note this fact and be on hand promptly at the time.

The ninth annual meeting of this Society will be held in the City Council Chamber, at Columbus, O., on the first Wednesday, December, 2d 1874, commencing at 10 a. m., and continuing its session three days. J. M. Porter, Secretary, H. A. Smith, President.

SUBJECTS FOR DISCUSSION.

1. Preservation of the Pulps of Teeth.

a. Systemic conditions modifying treatment, and influencing its results.

b. The Least Injurious, and yet efficient agents for covering and protecting dental pulp and likewise promoting the formation of new (secondary) dentine.

c. Causes of failure.

2. Contour Fillings.

a. The conditions and circumstances demanding this kind of fillings.

b. Failures and their causes.

3. Status of Mechanical Dentistry.

a. Can the mechanical branch be rescued from its demoralized condition, and be raised to a higher, and professional standing?

b. The means of accomplishing this.

c. Has the time arrived for the separation of the operative or surgical, from the mechanical department?

d. Advantage and disadvantage of such separation to each department.

4. Practical Views and results of experience in the use of the various preparations of gold for filling teeth.

a. Cohesive Gold—Is it as much used now, as formerly?

b. Non-Cohesive Gold—Reasons for preferring it to cohesive.

5. The best and most skillful method of applying the rubber dam.

a. Practical demomstrations in its application.

6. Dental Hygeine.

Clinics for the diagnosis, prognosis and treatment of diseased conditions of the mouth and teeth, together with the exhibition of instruments and appliances, will be held from 2 o'clock till 5 on the second day.

F. H. Rehwinkle, J. H. Warner, and C. H, Harronn, Executive Committee.

The State Board of Dental Examiners, will meet at the Neil House, at twelve o'clock m., December 1st, 1874, W. P. Horton, Secretary, J. Taft, President.

### ATTENTION.

### THE DENTAL REGISTER,

### A Monthly Magazine devoted entirely to the Interest of

### THE DENTAL PROFESSION.

**Terms Reduced to $2 50 per Year. Single Numbers 25cts.**

**EDITED BY PROF. J. TAFT,**

**And Published by SPENCER. CROCKER & CO., Ohio Dental Depot.**

The volume commences in January and closes in December.

The REGISTER being issued monthly is always fresh, therefore indispensable to the practitioner who desires to keep posted up with the new inventions and improvements which are daily developed. Neither expense nor effort will be spared to give value and variety to its contents. Articles will be illustrated with well executed engravings whenever they will add to the interest or assist in explanation.

Its extensive circulation and frequency of issue make it one of the BEST DENTAL ADVERTISING MEDIUMS in the United States.

All subscriptions must begin with January or July.

### RATES OF ADVERTISING.

| | 1 Mo. | 3 Mo. | 6 Mo. | 1 Year. | | 1 Year. | |
|---|---|---|---|---|---|---|---|
| 1 page.... | $12 00 | $25 00 | $40 00 | $75 00 | Inserted pages must | 1st page. | $50 00 |
| ½ page ... | 8 00 | 15 00 | 25 00 | 40 00 | be plain paper and | 2d page. | 37 50 |
| ¼ page ... | 5 00 | 10 00 | 15 00 | 25 00 | black ink. | 3d page. | 33 33 |
| | | | | | | 4th page. | 25 00 |

Local or special notices, five lines or less, will be inserted for $3 each insertion: over five lines, 50 cts. per line

All advertising bills payable in advance, or at furthest, after the issue of the first number containing the advertisement. All transient advertisements to be paid for in advance.

☞ CONTRIBUTIONS SOLICITED.

Exclusively Editorial Communications should be addressed to

**DR. J. TAFT, EDITOR.**

The latest number of the REGISTER may be ordered with or without other goods at any time, and all letters should be addressed to

**SPENCER, CROCKER & CO., OHIO DENTAL DEPOT,**

Cincinnati, Ohio.

